# Deep Reinforcement Learning-Based Coordinated Beamforming for mmWave Massive MIMO Vehicular Networks

**DOI:** 10.3390/s23052772

**Published:** 2023-03-03

**Authors:** Pulok Tarafder, Wooyeol Choi

**Affiliations:** Department of Computer Engineering, Chosun University, Gwangju 61452, Republic of Korea

**Keywords:** deep reinforcement learning, vehicular network, massive MIMO, beamforming, mmWave

## Abstract

As a critical enabler for beyond fifth-generation (B5G) technology, millimeter wave (mmWave) beamforming for mmWave has been studied for many years. Multi-input multi-output (MIMO) system, which is the baseline for beamforming operation, rely heavily on multiple antennas to stream data in mmWave wireless communication systems. High-speed mmWave applications face challenges such as blockage and latency overhead. In addition, the efficiency of the mobile systems is severely impacted by the high training overhead required to discover the best beamforming vectors in large antenna array mmWave systems. In order to mitigate the stated challenges, in this paper, we propose a novel deep reinforcement learning (DRL) based coordinated beamforming scheme where multiple base stations serve one mobile station (MS) jointly. The constructed solution then uses a proposed DRL model and predicts the suboptimal beamforming vectors at the base stations (BSs) out of possible beamforming codebook candidates. This solution enables a complete system that facilitates highly mobile mmWave applications with dependable coverage, minimal training overhead, and low latency. Numerical results demonstrate that our proposed algorithm remarkably increases the achievable sum rate capacity for the highly mobile mmWave massive MIMO scenario while ensuring low training and latency overhead.

## 1. Introduction

With the recent advancements in 5G, it is not ambitious to expect that 5G will enable 1000× more data traffic than the widely established current 4G standards [1,2]. Foreseeing the rise is users in increased traffic demands, facilitating these massive users and serving great quality cellular networks require high-frequency waves. Recently, millimeter wave (mmWave) communication has attracted significant interest in designing 5G wireless communication systems owing to its advantages in reducing spectrum scarcity and enabling high data speeds [3]. The range of the mmWave frequency band lies between 30 GHz to 300 GHz. However, the higher frequency travels very short distances due to their physical limitations in the spectrum and demonstrates high path loss [4]. Consequently, higher frequencies require smaller cellular cells to overcome the challenges such as path loss and blockage [5]. The massive multiple-input multiple-output (mMIMO) can use hundreds of antennas simultaneously to propagate signal in the same time-frequency resource and serve tens of users at the same time [6]. The mMIMO techniques can be utilized to perform highly directional transmissions thanks to the short wavelength of mmWave, which makes it physically feasible to equip a lot of antennas at the transceiver in a cellular network and can significantly improve network capacity [7,8]. Under a fairly generic channel model that considers poor channel estimation, pilot contamination, path loss, and terminal-specific antenna correlation, large-scale antenna systems significantly increase the achievable upload and download rate [9]. In situations with rapid changes in propagation, large-scale antenna systems can reliably offer high throughput on both the forward and the reverse link connections [10].

Vehicles are getting more sensors as driving becomes increasingly automated, resulting in increasingly higher data rates. Beamforming in mMIMO makes it possible to serve distance users with mmWave, even users that are not stationary. Therefore, the practical method for large bandwidth connected automobiles is mmWave mMIMO communication [11]. As a result, mmWave mMIMO systems can serve mobile vehicles effectively, considering the proper beam is selected. Due to the fundamental differences between mmWave communications and current microwave-based communication technologies (e.g., 2.4 GHz and 5 GHz), the mmWave systems present difficulties, such as a high sensitivity to shadowing and a significant signal attenuation [12]. In this paper, in order to overcome these issues and allow mMIMO environments where a highly non-stationary active user is present, we introduce a coordinated beamforming scheme utilizing deep reinforcement learning (DRL) to select the optimal beam for a vehicular communication system. First, a deep Q-network (DQN) algorithm is created to handle the beam selection problem as a Markov decision process (MDP). Then, by ensuring that the limitations of the beam selection matrix are met, our goal is to choose the best beams to maximize the sum rate for the user served.

### 1.1. Related Works

There have been few standard traditional approaches for beamforming or beam selection. In [13], Gao et al. followed an exhausting search approach for beamforming which demonstrates very high complexities in the system. On the other hand, Pal et al. [14] followed a different approach that iterated through the users and beams to determine the best possible beamforming matrices. This approach is also executed with high complexity algorithm.

On the other hand, deep learning (DL) based approaches show promising results in terms of application complexity and viability. Alkhateeb et al. [15] derived a high mobility supported mmWave mMIMO-based DL-enabled coordinated beamforming scheme for an outdoor scenario. To formulate their design, they utilized distributed base stations (BSs) simultaneously to serve a mobile user. They predicted the optimal beams using the traditional DL approach and compared the achievable rate performance of their DL method with the optimal achievable rate of beamforming. Zhang et al. [16] proposed a multi-user mMIMO coordinated beamforming scheme for heterogeneous networks (HetNets) focusing on energy efficiency (EE) based on the convolutional neural network (CNN) approach. They designed and used a multi-user huge MIMO HetNets optimization challenge to maximize EE with less complexity and compute delay. In order to accomplish end-to-end autonomous beamforming [17], introduced a constrained deep neural network-based beamforming technique. This method uses a neural network in place of the beamforming matrices used in conventional beamforming.

In [18], in-depth experiments for coordinated multipoint transmission at 73 GHz were carried out in a downtown Brooklyn urban open square setting. The analysis showed that serving a user jointly at the same time by many BSs can achieve a considerable coverage improvement. Moreover, another work on BS coordination, where a user is concurrently given access by many BSs, may be used to generate a significant coverage increase and is demonstrated by Maamari et al. in an analysis of the performance of heterogeneous mmWave cellular networks in [19]. Gupta et al. in [20] investigated the scope of a minimum of one line of sight (LOS) case when the users are served with LOS connections. The results showed that the density of coordinating BSs should scale with the square of the blockage density in order to maintain the same LOS connection. Although [18,19,20] established how BS coordination significantly increased coverage, they lack the analysis of producing coordinated beamforming vectors.

In order to enable high-speed, long-range, and reliable transmission in mmWave 60 GHz wireless personal area networks, Wang et al. [21] introduced a beamforming approach applied in the media access control (MAC) layer on top of various physical layer (PHY) designs. Ref. [11] suggested a new strategy to lower the overhead for beam alignment by utilizing dedicated short-range communication (DSRC) and/or sensor information as side information. Afterward, they provided detailed examples of how to leverage location data from DSRC to lessen the overhead of beam alignment and tracking in mmWave vehicle-to-everything (V2X) applications. Conversely, Ref. [22] proposed an algorithm to jointly optimize the beamforming vectors and power allocation for reconfigurable intelligent surface (RIS)-based applications. Lin et al. in [23] formulated solutions on the joint design and optimization of beamforming for hybrid satellite-terrestrial relay networks with RIS support, and in [24] proposed another methodology for joint beamforming for mmWave non-orthogonal multiple access (NOMA). Furthermore, the author also investigated secure energy-efficient beamforming in multibeam satellite systems in [25].

On the other hand, Va et al. [26] proposed a multipath fingerprint database using the vehicle’s position (for example, as determined by GPS) to gain information on probable pointing directions for accurate beam alignment. The power loss probability is a parameter used in the method to measure misalignment precision and is used to enhance candidate beam selection. Moreover, two candidate beam selection techniques are created, one of which uses a heuristic, and the other aims to reduce the likelihood of misalignment. Cao et al. [27] proposed a latency reduction scheme for vehicular network relay selection. In addition, Zhou et al. [28] proposed a DQN-based algorithm to train and determine the optimal receiver beam direction with the purpose of maximizing average received signal power.

However, there are various drawbacks to designing beamforming vectors by utilizing the stated approaches, such as solely based on location data and received signal power. First, narrow-beam systems may not function effectively with position-acquisition sensors like GPS because of their poor precision, which is typically in the range of meters. Second, these technologies are unable to handle indoor applications since GPS sensors perform poorly inside structures. In addition, the beamforming vectors depend on the environment’s shape, obstructions, and so on. Furthermore, received signal power can experience severe penetration power loss because of the vehicle’s metal body. In this paper, we aim to utilize a DRL-based coordinated approach where we do not encounter the declared challenges and exhibit better results.

### 1.2. Contribution

In this paper, for highly mobile mmWave applications, we provide a novel DRL approach for highly mobile mmWave communication architecture. As part of our suggested method, a coordinated beamforming system is used, in which a number of BSs concurrently provide access to a single non-stationary user. In this approach, a DRL network exclusively utilizes beam patterns and learns how to anticipate the BSs beamforming vectors from the signals obtained at the scattered BSs. The idea behind this is that the propagated waves collectively acquired at the scattered BSs indicate a distinctive multi-path signature of both the user position and its surroundings. There are several benefits to the suggested approach. First, the suggested technique can accommodate not only LOS but non-LOS (NLOS) framework without the need for specialized position-acquiring devices because beamforming prediction is based on the uplink received signals rather than position data. Second, only received pilots, which may be retrieved with minimal overhead training, are needed for the determination of the best beams. Furthermore, because the DRL model trains and responds to any environment, it does not need any training before deployment in the suggested system. The proposed model also inherits coordination coverage and reliability improvements since it is coupled with the coordinated beamforming mechanism. Even though some DRL-based beamforming solutions exist, to the best of our knowledge, no prior work addressed a coordinated beamforming solution by leveraging DRL where multiple BSs serve one single mobile user jointly to achieve the highest possible data rate. The contributions of the proposed beamforming scheme are summarized as follows:

- We develop a simple coordinated beamforming scheme where several BSs employ RF beamforming and are connected to a central cloud processing unit that uses baseband processing, which serves a mobile user at once. To increase the platform’s effective achievable rate, we define a training and design issue for the central baseband processing and for BSs RF beamforming vectors. The trade-off between the beamforming training overhead and the achievable sum rate using the proposed beamforming vectors is taken into account when determining the effective achievable rate for highly mobile mmWave systems.
- For the selected system, we construct a fundamental coordinated beamforming technique that relies on uplink training for creating the RF and baseband beamforming vectors. The BSs choose their RF beamforming vectors from a predetermined codebook as part of this baseline approach. The baseband beamforming is then designed by a central processor to guarantee consistent incorporation by the user. We demonstrate that the standard beamforming technique achieves the best attainable rates in a few unique but crucial situations.
- We introduce a system operation of machine learning modeling of a unique combined DRL and coordinated beamforming solution. In this approach, we incorporate a reverse autoencoder owing to its capability to handle raw data seamlessly so that it can reproduce the input data as closely as possible as a neural network for our DRL model and solve a coordinating beamforming problem. The main concept of the suggested technique is to anticipate the RF beamforming vectors of the coordinating BSs using just beam patterns, i.e., with very little training overhead. The proposed approach also enables minimal coordination overhead harvesting of coordinated beamforming improvements with wide coverage and low latency, making the method a viable solution for highly mobile mmWave applications.

## 2. System Model

**Figure 1:**
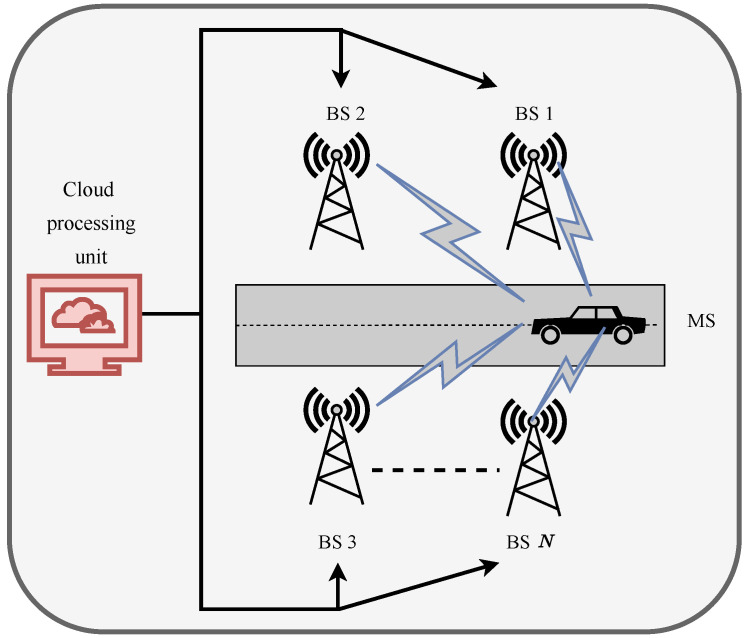
Downlink mmWave mMIMO vehicular beamforming system.

In this section, we discuss the chosen frequency-selective coordinated mmWave system and channel models for our DRL-based coordinated beamforming scheme, where for this designated system and channel model, the DRL model optimizes the beam selection from a set of candidate beams by utilizing the exploration and exploitation strategy of the DRL. We analyze a mmWave-enabled vehicular communication architecture shown in Figure 1, where *N* BSs are concurrently providing service to one mobile station (MS). Each BS is equipped with *M* a number of antennas, and each BS is linked to a central processing unit in the cloud. In the interests of simplicity, we assume that each BS utilizes analog-only beamforming with networks of phase shifters and has a single RF chain [29]. In this paper, we use the assumption that the MS is equipped with only one antenna.

The signals are precoded using a N× 1 digital precoder $f_{k}\in C^{N\times 1}$ for subcarrier *k*, k=1,⋯, *K*. The frequency domain signals are then converted into the time domain using *N* *K*-point inverse fast Fourier Transforms (IFFTs). Afterward, each BS *n* performs a time-domain analog beamforming and then transmits the resulting signal. At the receiver end, the received signal is converted to the frequency domain using a *K*-point FFT, presuming perfect synchronization of frequency and carrier offset. The received signal at *k*th subcarrier at *n*th BS is denoted by

(1) $$y_{k}=\sum_{n=1}^{N}h_{k,n}^{T}x_{k,n}+n_{k},$$

where $x_{k,n}$ is the transmitted complex baseband signal, $h_{k,n}$ is the M× 1 channel vector between the MS and BS, $n_{k}\in C^{M\times 1}$ is the received noise at the BS with independent and identically complex (i.i.c.) additive white Gaussian noise (AWGN) distribution with zero mean and variance $\sigma^{2}$.

We consider a *L* clustered geometric wideband model for our mmWave cellular channel [30,31,32]. For each cluster *l*, it is assumed that l=1,⋯, *L* contributes one ray with a temporal delay ${Ø}_{l}\in R$, and azimuth/elevation angles of arrival (AoA) is $\theta_{l},\phi_{l}$. Let p(τ) be a pulse shaping function for $T_{S}$-spaced signaling assessed at τ seconds, and let $\rho_{n}$ signify the path-loss between the user and the *n*th BS [33]. The delay-d channel vector in this model $h_{d,n}$ between the user and the *n*th BS can be expressed as

(2) $$h_{d,n}=\sqrt{\frac{M}{\rho_{n}}}\sum_{l=1}^{L}\alpha_{l}p\left(\tau \right)\left(dT_{s}-\tau_{l}\right)a_{n}\left(\theta_{l},\phi_{l}\right)$$

where $\alpha_{l}$ is the gain, $a_{n}\left(\theta_{l},\phi_{l}\right)$ is the array response vector ($\theta_{l}$ = azimuth angle, $\phi_{l}$ = elevation angle) of the *n*th BS. Considering the delay-d channel in (2), for subcarrier *k*, our frequency domain channel vector $h_{k,n}$ can be formulated as

(3) $$h_{k,n}=\sum_{d=0}^{D-1}h_{d,n}\exp\left(-j\frac{2\pi k}{K}d\right).$$

Our adopted block-fading channel model ${\left\{h_{k,n}\right\}}_{k=1}^{K}$ is considered to remain constant throughout the channel coherence time, and it is dependent on the user the mobility and the channel multi-path components [34].

## 3. Coordinated Beamforming

**Figure 2:**
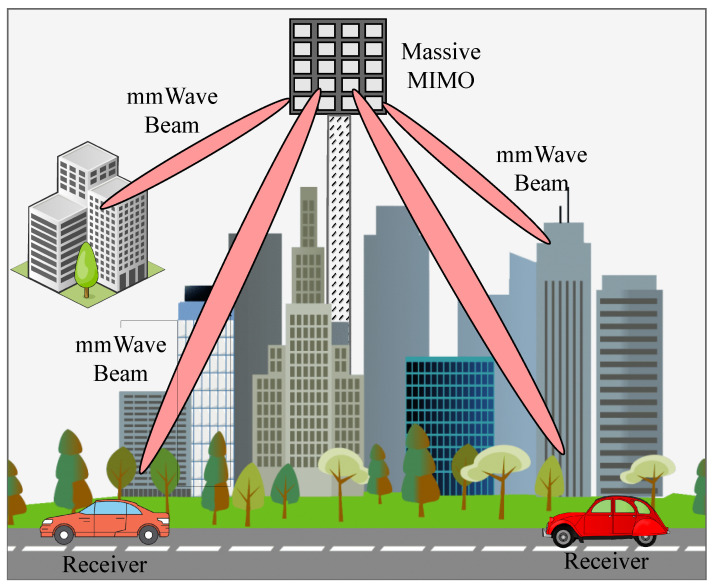
Overview of mMIMO beamforming.

In this chapter, we introduce a baseline DRL coordinated beamforming approach for a highly mobile vehicular mmWave communication system as shown in Figure 2. To present the proposed solution, we first describe the problem formulation, then derive the novel DRL-based approach for beamforming. In this chapter, we also present the environment setup, dataset generation, simulation parameters, and performance analysis for our proposed scheme.

### 3.1. Problem Statement

For a vehicular mmWave based 5G network, serving any user or MS is challenging because of the dynamic and varying environment characteristics. When signal interference, fading effect, and network congestion are considered, which we subsequently describe as the environment dynamics [35], it becomes much more complicated to serve the receiver end by maintaining eMBB, mMTC, and URLLC standards. Considering the time-varying environment of wireless communication, a DRL-based beamforming scheme is most appropriate. In the case of DL-based approaches, they struggle to show promising results while dealing with the stated time-varying environments because they lack the functionality of learning by good or bad actions. To achieve the highest level of sum rate, reduce the overhead, and tackle the large RF beamforming vector arrays, an adaptive beam selection approach such as DRL is best suited for this specific task. With this motivation, in this paper, we exploit the DRL’s capability of tackling varying environments to maximize the achievable data rate by selecting the optimal beam for mmWave vehicular networks in a coordinated approach.

In this paper, considering a set of beamforming vectors ${\left\{f_{n}^{BF}\right\}}_{n=1}^{N}$, our focus is to formulate a beam selection matrix to optimize the achievable downlink rate of the mmWave vehicular beamforming system. The user’s maximum achievable rate can be derived as

(4) $$R=\frac{1}{K}\sum_{k=1}^{K}\log_{2}\left(1+SNR{\left|\sum_{n=1}^{N}h_{k,n}^{T}f_{n}^{BF}\right|}^{2}\right).$$

### 3.2. Drl-Based Coordinated Beamforming Framework

We propose a DRL framework that utilizes DQN to train and optimize the beam selection assignment. Typically, the DQN technique consists of an environment and an agent using a deep neural network (DNN). The agent, the same as BS in this study, engages with the environment before performing any action. In the beginning, the agent starts exploring the environment, moving from one state to another. At that point, it needs more information about the environment. As the agent explores the environment, it gathers information and starts to take action by exploiting the environment with the help of the reward function. In any timestep *t*, if the current state is $S_{t}$, the agent will receive an immediate reward $R_{t}$ assessing the performed action $A_{t}$ using the DNN. The agent also gets to take the next state $S_{t+1}$ as input from the environment in the same timestep. Depending upon the performed $A_{t}$, the agent receives a reward $R_{t}$. If the action taken can achieve a reasonable sum rate, then the agent will also receive a good $R_{t}$. The agent gains knowledge of its surroundings and develops an ideal beam selection assignment strategy by foreseeing future events. The DNN algorithm learns this policy π at each timestep as it continues to move forward with the next timesteps. We formulate our state, action, and reward functions as follows:

- State: We utilize the channel matrices for all the BSs as the state of our environment. The complex channel matrices are constructed incorporating the bandwidth, user position, noise figure, and noise power. If the environment has *Z* states each having *V* number of beams, then, the state space with Z×V can be represented as $S=\tilde{S_{1}},\tilde{S_{2}},\tilde{S_{3}},\cdots$, $\tilde{S_{Z}}$.
- Action: The goal of the agent is to assign a beam for serving from the action space *A*. At each episode for a set of *S*, the agent has to take Z∈A actions while maintaining one action per *V* elements from the *S*. Out of the Z×V, the target of the agent is choosing a beam that will maximize the data rate.
- Reward: In our reward function, we first derive the data rate for each channel as follows.
  (5) $$R_{r}=\log_{2}\left(1+SNR{\left|\sum_{n=1}^{N}h_{k,n}^{T}f_{n}^{BF}\right|}^{2}\right).$$
  For every action the agent takes, we calculate the data rate of the chosen action and feed it as the reward value. Our aim is to acquire the highest possible cumulative reward $R_{\max}$ as it obtains reward for each action, according to
  (6) $$R_{\max}=\arg\max\sum_{k=1}^{K}\log_{2}\left(1+SNR{\left|\sum_{n=1}^{N}h_{k,n}^{T}f_{n}^{BF}\right|}^{2}\right).$$

**Figure 3:**
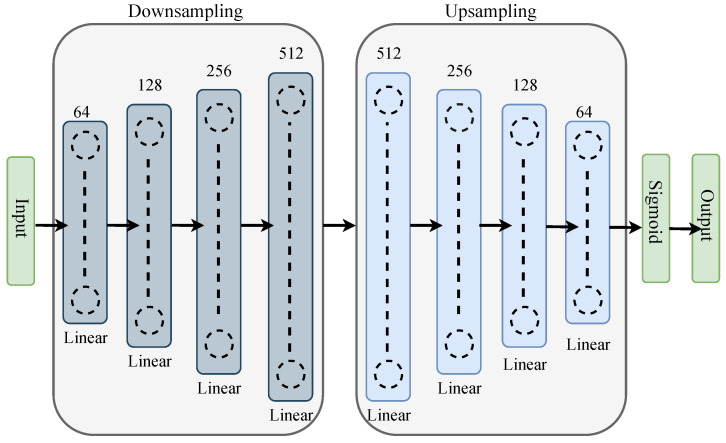
Proposed DNN architecture.

With this state, action, and reward function, we propose the DNN architecture as the policy controller for the beam selection, as shown in Figure 3. The DNN takes the place of the Q-table and calculates the Q-values for each environment state-action pair. Deriving probabilities for each beam selection for each state space is the primary objective of the DNN, and this probability can be defined by Q(S,A) of the DQN algorithm. We select the best beam out of *V* = 64 candidate beams, coordinately with 4 BSs.

### 3.3. Reverse Autoencoder

Autoencoder is a neural network that can be taught to reconstruct their input [36]. It is a particular kind of neural network that is primarily developed to compress and meaningfully represent the input before decoding it back so that the reconstructed input is as similar to the original as possible [37]. Moreover, the autoencoder can handle the raw input data without any difficulties and is viewed as a component of the unsupervised learning model [38]. The autoencoder consists of three main components, encoder, code, and decoder. In addition, for autoencoders, the number of neurons decreases as we go deep down the hidden layers. However, it increases [39] in reverse autoencoder. We resort to the newly introduced reverse autoencoder for the DNN segment of our DQN model.

In the encoder, the input layer starts with 2${}^{c}$ neurons, and the next hidden layers are followed by 2${}^{c+p}$ neurons. In this paper, we start the hidden layers with c= 5, and *p* refers to the position of the layer. For the code layer, we use the value of c+p= 9 for the code layer. The decoder portion ends with an output layer and is the exact opposite of the encoder portion. Because the layers are placed one on top of the other like a sandwich, this form of structure is referred recognized as a stacked autoencoder. Additionally, each layer in the autoencoder has its own ReLu activation function.

## 4. Performance Evaluation

In this section, we evaluate the proposed DRL-based coordinated beamforming approach in different case studies by comparing it with traditional DL architecture [15]. In a multicell mmWave mMIMO downlink scenario, a large uniform planar array (UPA) is installed on a BS. In this paper, we select 4 BS with 32×8 UPA, resulting in M=256 antenna arrays for each BS.

For our methodology, we used the popular publicly available DeepMIMO [40] dataset generated by the Wireless InSite [41]. The dataset contains the generated beamforming vectors or predetermined codebooks, denoted as $f_{n}^{BF}$. These generated codebooks are the beamforming defining vectors. Along with $f_{n}^{BF}$, we also use their corresponding channel matrices as depicted as $h_{k,n}$ in our proposed scheme for defining states and rewards as discussed in Section 3.2 for optimizing the optimal beamforming vectors.

We used the outdoor scenario of two streets and one intersection with a mmWave communication operating at 60 GHz. We aim to serve the MS with the best beam, coordinating with 4 BSs. For this adopted scenario, 4 BSs are equipped on the top of 4 lamp posts to concurrently provide beam coverage for one MS coordinately. The lamps are located 60 m away, side by side. Every BS is installed on the 6 m elevation having 32 × 8 antenna elements. The MS is incorporated with a single antenna on top of the vehicle. During the uplink training, we assumed a transmit power of 30 dBm for the MS. The adopted DeepMIMO parameters for dataset generation and the simulation parameters used in this work are summarized in Table 1 and Table 2, respectively.

### 4.1. Training

The proposed DNN is gradually trained using a set of training data for each episode. For every state space *S*, the state action pair is formulated using the ϵ-greedy policy in accordance with the output probabilities of DNN. An episode is considered complete when all state space has been processed by the DNN. For every state space, the exploitation policy [42] or the policy for taking action can be represented as

(7) $$\begin{array}{c} a_{t}^{l}=\left\{\begin{array}{cc} argmax\;Q(S_{t},A_{t}^{l}) & \mathrm{if}\;\epsilon <\epsilon_{th},\;\epsilon_{th}\in \left(0,1\right]\\ random\;action[1,V] & otherwise \end{array}\right.,\\ \begin{array}{cccc} \; & \; & & \forall l=1,2,\ldots ,Z\in A,\\ \; & \; & & \forall t=1,2,\ldots ,ins. \end{array} \end{array}$$

**Figure 4:**
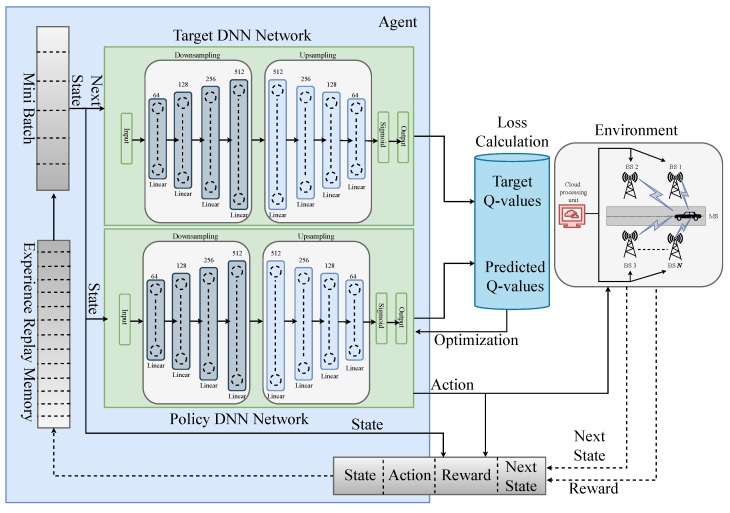
Proposed DRL Framework.

After executing $a_{t}^{l}$, the agent will receive the rewards according to (5) and the next state space $S_{t+1}$. Afterward, we first determine the loss and then tweak the DNN’s parameters using back-propagation to train our model. We take an approximation of the optimal Q*-values for each state-action pair for $S_{t+1}$ from a separate DNN termed the target DNN [43] in order to compute the loss. The policy DNN’s settings are used to initialize the target DNN, which is identical to it. Consequently, for the target DNN input, we use the next state space $S_{t+1}$ as the input, and finally, the agent chooses optimal $Q^{*}$-values greedily from the output. We add experience replay memory (ERM) to the DQN to help the optimal policy converge more steadily [44]. The agent first explores its environment while saving its current states, actions, rewards and next states $\left(S_{t},A_{t},R_{t},S_{t+1}\right)$ as a tuple in the ERM. The agent then trains the policy DNN using a small batch of tuples from the ERM. Each training set of data continues to be updated in the ERM. We summarize the system architecture and working principles of our model in Figure 4 and Algorithm 1.
**Algorithm 1** Proposed deep *Q*-learning algorithm1:Initialize policy, target DQN with random *w*, $w^{\prime }$2:Initialize ϵ3:**for** episode **do**4:    **for** instance **do**5:        Select a channel matrix and add it to action space $A_{t}$ for present state space $S_{t}$6:        Observe immediate reward $R_{t}$, next state space $S_{t+1}$7:        Put $\left(S_{t},A_{t},R_{t},S_{t+1}\right)$→ ERM8:        Form random sample mini batch of $\left(S_{t},A_{t},R_{t},S_{t+1}\right)$ from ERM9:        **for** each tuple in mini batch **do**10:           Calculate *Q*-values11:           Approximate $Q^{*}$-values using target DNN12:           Compute loss from *Q* and $Q^{*}$13:           Optimize *w* of policy DNN with Adam optimizer14:    $w^{\prime }\leftarrow w$ after all time steps**Ensure:** $R_{r}\approx R_{\max}$

In the training phase of our model, we used Adam optimizer [45] with a learning rate of 0.0005. The DRL model minimizes the error of our training in the DNN using the Smooth${}_{L1}$ loss function [46,47]. If we have a batch of size *B*, the unreduced loss for two data points (u,w) can be described as

(8) $$\mathrm{loss}\left(u,w\right)={\left\{loss_{1},\ldots ,loss_{B}\right\}}^{T},$$

where in any loss instance b∈B,

(9) $${\mathrm{loss}}_{b}=\left\{\begin{array}{cc} 0.5{\left(u_{b}-w_{b}\right)}^{2}/\beta & \mathrm{if}\left|u_{b}-w_{b}\right|<\beta \\ \left|u_{b}-w_{b}\right|-0.5\times \beta & \mathrm{otherwise}\; \end{array}\right..$$

### 4.2. Performance Analysis

**Figure 5:**
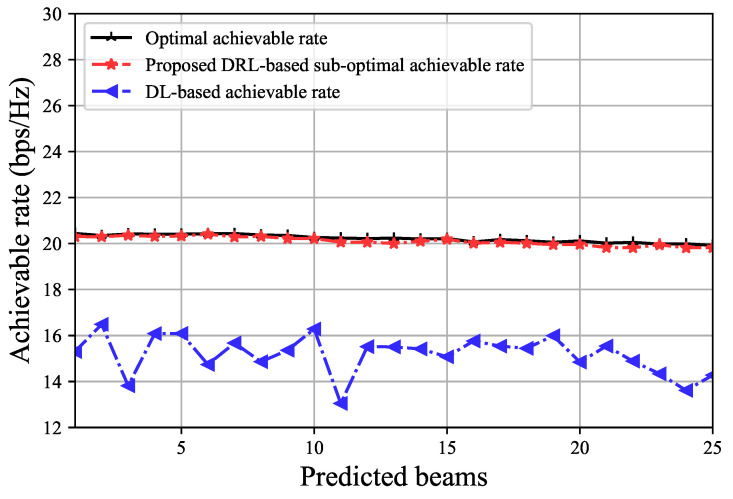
A comparison of effective achievable rates without overhead consideration.

In this subsection, we will evaluate our achieved performance in terms of sum rate and will compare our rate with the traditional ML approach. Figure 5 represents the performance analysis of our proposed model having 3 performance matrices. We plot an effective achievable rate based on our DRL, conventional DL, and optimal data rate. Even though the utilization of the reverse autoencoder in the DRL model incorporated higher computation complexity and time complexity in the learning phase of the model. However, the delays we verified in our simulations are slight in the considered system environment. In addition, we confirmed that the performance degradation due to the delays is insignificant in the simulation results. It is clear that our proposed DRL outperforms the traditional DL model by a large margin and demonstrates suboptimal performance. In this figure, we did not consider any beam training or latency overhead. For vehicular mmWave communication, when the user is mobile, one of the most viable communication overheads is velocity because the connectivity between the BS and the user gets affected by the velocity. For fast-moving users, it needs fast beam switching from the BS, otherwise, because of the delay, the user might not get service on time from the BS as it moves away from its current position.

**Figure 6:**
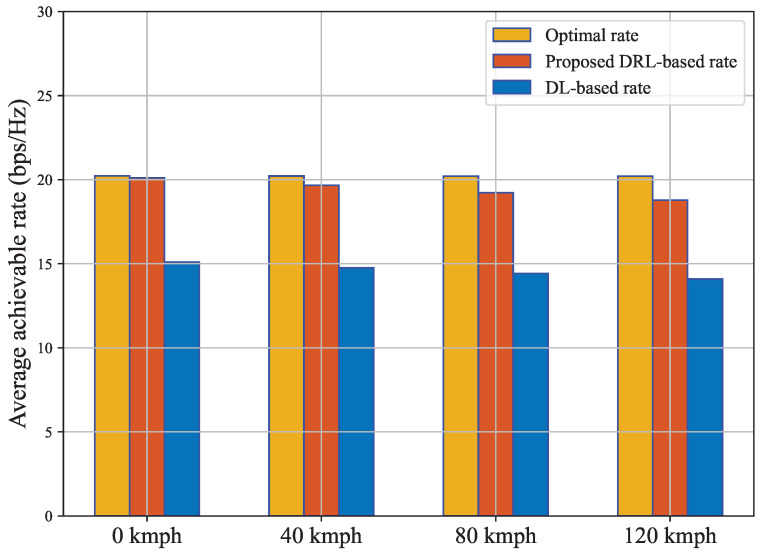
A comparison of effective achievable rate including overhead consideration.

In Figure 6, we compare DRL and DL-based beamforming performance with the optimal beamforming performance by incorporating overhead. More specifically, we compared our DRL-based achievable sum rate for all the 3 overhead speed side by side. The performance was similarly very consistent throughout the plot, and the achievable rate of declination due to the increased overhead was negligible. In this stage, we consider the 64-beam training overhead, with coherence time at 40 kmph at first. It is visible that, even though our suboptimal performance experienced a slight decrease, the DRL beamforming achievable rate is still significantly higher than the DL approach. We also compared the achievable rate versus different user positions at 80 kmph and 120 kmph in the same Figure 6. The results followed similar trends. Our DRL-based approach outperformed the DL approach by a large margin and demonstrated suboptimal performance. As the user position moved, the achievable rate saw a slight but steady decrease over the period. However, for the traditional DL-based approach, the performance was inconsistent.

**Figure 7:**
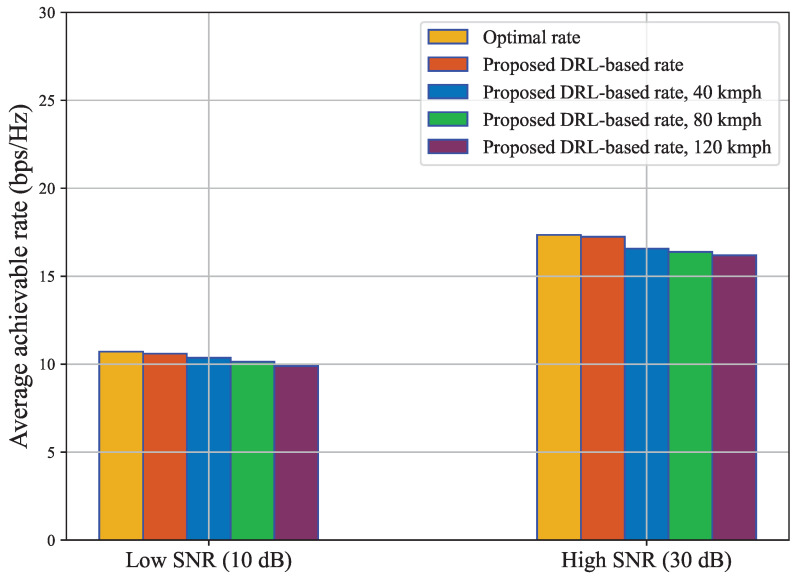
A comparison of effective achievable rate including overhead consideration at high and low SNR.

We also compare the performance of our proposed DRL scheme by varying SNR as shown in Figure 7. It is demonstrated how the performance of our model varies at two different SNR levels, which are low SNR at 10 dB and high SNR at 30 dB. The previous results containing 38.65 dB SNR portrayed higher results. We confirmed that after the SNR was reduced to 30 dB, the initial performance dropped by 14.27% in terms of the average sum rate for our DRL method. In addition, we have illustrated the performance of our DRL model at an SNR of 10 dB in this figure. It is noticeable that, for another 20 dB of SNR drop, the performance declined by another 38.51%.

**Figure 8:**
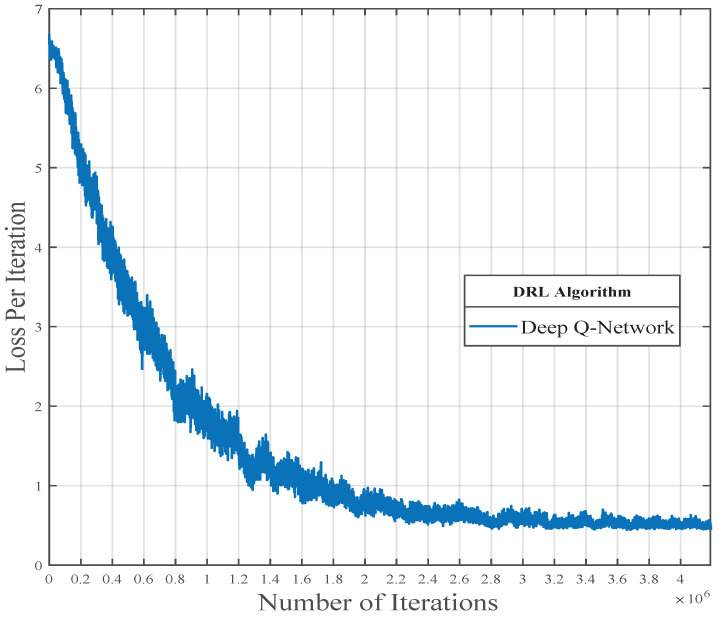
Loss convergence plot for the proposed DQN-based coordinated beamforming.

Furthermore, in Figure 8, we have illustrated the convergence of our proposed algorithm. The achievable sum rate converges with the time step *t* in terms of loss. After approximately $3.2\times 10^{6}$ iterations, we confirmed that our model converged successfully. Overall, the performance of our model significantly rises as the SNR increases. Our proposed DRL architecture is robust and flexible in various conditions, such as different SNRs and different velocities.

## 5. Conclusions

In this paper, we propose a sub-optimal beam selection scheme with DRL that enables high mobile applications in mmWave mMIMO systems. The key idea is to utilize the powerful exploration-exploitation strategy of DRL to derive the optimal beam selection policy by learning the mapping of the omni-received uplink pilot and sub-optimal beam mapping. Our proposed scheme guarantees achievable sum rate performance close to optimal, even if it requires a small training overhead and beam overhead. In addition, the proposed scheme ensures reliable coverage and shorter latency while serving the beam towards the highly mmWave mobile user end.

## Figures and Tables

**Table 1:** Dataset parameters.

Parameters	Values
Scenario	O1_60
Active BS	3,4,5,6
Receivers	R1000–R1300
Frequency band	60 GHz
Bandwidth	500 MHz
Number of OFDM subcarriers	1024
Subcarrier limit	64
Number of paths	5
BS antenna shape	1×32×8
Receiver antenna shape	1×1×1

**Table 2:** Simulation parameters for the DRL model.

Parameters	Values
Beams per BS distribution	16
Total beams	64
Transmit power	30 dBm
Learning rate (LR)	0.0005
Discount factor (γ)	0.999
Epsilon (ϵ)	[1, 0.1, 0.001]
Batch size	96
Number of episodes	250
Data instances	200

## References

[1] Saghezchi F.B., Rodriguez J., Mumtaz S., Radwan A., Lee W.C., Ai B., Islam M.T., Akl S., Taha A.E.M. (2015). Drivers for 5G: The ‘pervasive connected world’. Fundamentals of 5G Mobile Networks.

[2] Chen T., Matinmikko M., Chen X., Zhou X., Ahokangas P. (2015). Software defined mobile networks: Concept, survey, and research directions. IEEE Commun. Mag..

[3] Boccardi F., Heath R.W., Lozano A., Marzetta T.L., Popovski P. (2014). Five disruptive technology directions for 5G. IEEE Commun. Mag..

[4] Busari S.A., Huq K.M.S., Mumtaz S., Dai L., Rodriguez J. (2018). Millimeter-Wave Massive MIMO Communication for Future Wireless Systems: A Survey. IEEE Commun. Surv. Tutorials.

[5] Li Q., Niu H., Papathanassiou A.T., Wu G. (2014). 5G Network Capacity: Key Elements and Technologies. IEEE Veh. Technol. Mag..

[6] Larsson E.G., Edfors O., Tufvesson F., Marzetta T.L. (2014). Massive MIMO for next generation wireless systems. IEEE Commun. Mag..

[7] Ghosh A., Thomas T.A., Cudak M.C., Ratasuk R., Moorut P., Vook F.W., Rappaport T.S., MacCartney G.R., Sun S., Nie S. (2014). Millimeter-Wave Enhanced Local Area Systems: A High-Data-Rate Approach for Future Wireless Networks. IEEE J. Sel. Areas Commun..

[8] Rusek F., Persson D., Lau B.K., Larsson E.G., Marzetta T.L., Edfors O., Tufvesson F. (2013). Scaling Up MIMO: Opportunities and Challenges with Very Large Arrays. IEEE Signal Process. Mag..

[9] Hoydis J., ten Brink S., Debbah M. (2013). Massive MIMO in the UL/DL of Cellular Networks: How Many Antennas Do We Need?. IEEE J. Sel. Areas Commun..

[10] Marzetta T.L. (2010). Noncooperative Cellular Wireless with Unlimited Numbers of Base Station Antennas. IEEE Trans. Wirel. Commun..

[11] Choi J., Va V., Gonzalez-Prelcic N., Daniels R., Bhat C.R., Heath R.W. (2016). Millimeter-Wave Vehicular Communication to Support Massive Automotive Sensing. IEEE Commun. Mag..

[12] Tarafder P., Choi W. (2022). MAC Protocols for mmWave Communication: A Comparative Survey. Sensors.

[13] Gao X., Dai L., Chen Z., Wang Z., Zhang Z. (2016). Near-Optimal Beam Selection for Beamspace MmWave Massive MIMO Systems. IEEE Commun. Lett..

[14] Pal R., Srinivas K.V., Chaitanya A.K. (2018). A Beam Selection Algorithm for Millimeter-Wave Multi-User MIMO Systems. IEEE Commun. Lett..

[15] Alkhateeb A., Alex S., Varkey P., Li Y., Qu Q., Tujkovic D. (2018). Deep Learning Coordinated Beamforming for Highly-Mobile Millimeter Wave Systems. IEEE Access.

[16] Zhang Y., Zhang B., Wang H., Zhang T., Qian Y. Deep Learning-based Coordinated Beamforming for Massive MIMO-Enabled Heterogeneous Networks. Proceedings of the 2021 IEEE Global Communications Conference (GLOBECOM).

[17] Tao J., Wang Q., Luo S., Chen J. Constrained Deep Neural Network Based Hybrid Beamforming for Millimeter Wave Massive MIMO Systems. Proceedings of the ICC 2019—2019 IEEE International Conference on Communications (ICC).

[18] MacCartney G.R., Rappaport T.S., Ghosh A. Base Station Diversity Propagation Measurements at 73 GHz Millimeter-Wave for 5G Coordinated Multipoint (CoMP) Analysis. Proceedings of the 2017 IEEE Globecom Workshops (GC Wkshps).

[19] Maamari D., Devroye N., Tuninetti D. (2016). Coverage in mmWave Cellular Networks with Base Station Co-Operation. IEEE Trans. Wirel. Commun..

[20] Gupta A.K., Andrews J.G., Heath R.W. (2017). Macrodiversity in cellular networks with random blockages. IEEE Trans. Wirel. Commun..

[21] Wang J., Lan Z., Woo Pyo C., Baykas T., Sean Sum C., Rahman M., Gao J., Funada R., Kojima F., Harada H. (2009). Beam codebook based beamforming protocol for multi-Gbps millimeter-wave WPAN systems. IEEE J. Sel. Areas Commun..

[22] Niu H., Lin Z., Chu Z., Zhu Z., Xiao P., Nguyen H.X., Lee I., Al-Dhahir N. (2022). Joint beamforming design for secure RIS-assisted IoT networks. IEEE Internet Things J..

[23] Lin Z., Niu H., An K., Wang Y., Zheng G., Chatzinotas S., Hu Y. (2022). Refracting RIS-aided hybrid satellite-terrestrial relay networks: Joint beamforming design and optimization. IEEE Trans. Aerosp. Electron. Syst..

[24] Lin Z., Lin M., Wang J.B., De Cola T., Wang J. (2019). Joint beamforming and power allocation for satellite-terrestrial integrated networks with non-orthogonal multiple access. IEEE J. Sel. Top. Signal Process..

[25] Lin Z., An K., Niu H., Hu Y., Chatzinotas S., Zheng G., Wang J. (2022). SLNR-based secure energy efficient beamforming in multibeam satellite systems. IEEE Trans. Aerosp. Electron. Syst..

[26] Va V., Choi J., Shimizu T., Bansal G., Heath R.W. (2018). Inverse Multipath Fingerprinting for Millimeter Wave V2I Beam Alignment. IEEE Trans. Veh. Technol..

[27] Cao D., Zheng B., Ji B., Lei Z., Feng C. (2020). A robust distance-based relay selection for message dissemination in vehicular network. Wirel. Netw..

[28] Zhou X., Zhang X., Chen C., Niu Y., Han Z., Wang H., Sun C., Ai B., Wang N. (2022). Deep Reinforcement Learning Coordinated Receiver Beamforming for Millimeter-Wave Train-Ground Communications. IEEE Trans. Veh. Technol..

[29] Heath R.W., González-Prelcic N., Rangan S., Roh W., Sayeed A.M. (2016). An Overview of Signal Processing Techniques for Millimeter Wave MIMO Systems. IEEE J. Sel. Top. Signal Process..

[30] Rappaport T.S., Sun S., Mayzus R., Zhao H., Azar Y., Wang K., Wong G.N., Schulz J.K., Samimi M., Gutierrez F. (2013). Millimeter Wave Mobile Communications for 5G Cellular: It Will Work!. IEEE Access.

[31] Akdeniz M.R., Liu Y., Samimi M.K., Sun S., Rangan S., Rappaport T.S., Erkip E. (2014). Millimeter Wave Channel Modeling and Cellular Capacity Evaluation. IEEE J. Sel. Areas Commun..

[32] Samimi M.K., Rappaport T.S. Ultra-wideband statistical channel model for non line of sight millimeter-wave urban channels. Proceedings of the 2014 IEEE Global Communications Conference.

[33] Schniter P., Sayeed A. Channel estimation and precoder design for millimeter-wave communications: The sparse way. Proceedings of the 2014 48th Asilomar Conference on Signals, Systems and Computers.

[34] Va V., Choi J., Heath R.W. (2017). The Impact of Beamwidth on Temporal Channel Variation in Vehicular Channels and Its Implications. IEEE Trans. Veh. Technol..

[35] Sana M., De Domenico A., Yu W., Lostanlen Y., Calvanese Strinati E. (2020). Multi-Agent Reinforcement Learning for Adaptive User Association in Dynamic mmWave Networks. IEEE Trans. Wirel. Commun..

[36] McClelland J.L., Rumelhart D.E., PDP Research Group (1987). Volume 2: Explorations in the Microstructure of Cognition: Psychological and Biological Models. Parallel Distributed Processing.

[37] Bank D., Koenigstein N., Giryes R. (2020). Autoencoders. arXiv.

[38] Kingma D.P., Welling M. (2013). Auto-encoding variational bayes. arXiv.

[39] Ling C., Cao G., Cao W., Wang H., Ren H. (2021). IAE-ClusterGAN: A new Inverse autoencoder for Generative Adversarial Attention Clustering network. Neurocomputing.

[40] Alkhateeb A. DeepMIMO: A Generic Deep Learning Dataset for Millimeter Wave and Massive MIMO Applications. Proceedings of the Information Theory and Applications Workshop (ITA).

[41] Remcom Wireless InSite. http://www.remcom.com/wireless-insite.

[42] Rezwan S., Choi W. (2021). Priority-based joint resource allocation with deep q-learning for heterogeneous NOMA systems. IEEE Access.

[43] Sutton R.S., McAllester D., Singh S., Mansour Y. (1999). Policy gradient methods for reinforcement learning with function approximation. Adv. Neural Inf. Process. Syst..

[44] Zhang S., Sutton R.S. (2017). A deeper look at experience replay. arXiv.

[45] Kingma D.P., Ba J. (2014). Adam: A method for stochastic optimization. arXiv.

[46] Girshick R. Fast r-cnn. Proceedings of the IEEE International Conference on Computer Vision.

[47] SmoothL1Loss—PyTorch 1.13 Documentation. https://pytorch.org/docs/stable/generated/torch.nn.SmoothL1Loss.html.

